# Development of a Textile Nanocomposite as Naked Eye Indicator of the Exposition to Strong Acids

**DOI:** 10.3390/s17092134

**Published:** 2017-09-16

**Authors:** Isabel Pallás, Maria D. Marcos, Ramón Martínez-Máñez, Jose V. Ros-Lis

**Affiliations:** 1 Instituto Interuniversitario de Investigación de Reconocimiento Molecular y Desarrollo Tecnológico, 46022 Valencia, Spain; ispalra@etsid.upv.es (I.P.); mmarcos@qim.upv.es (M.D.M.); rmaez@qim.upv.es (R.M.-M.); 2 CIBER de Bioingeniería, Biomateriales y Nanomedicina (CIBER-BBN), 46022 Valencia, Spain; 3 Departamento de Química Inorgánica, Universitat de València, Doctor Moliner 50, Burjassot, 46100 Valencia, Spain

**Keywords:** sensor, indicator, mesoporous material, colour, strong acid, textile

## Abstract

Chemical burns, mainly produced by acids, are a topic of concern. A new sensing material for the detection of strong acids able to be incorporated into textiles has been developed. The material is prepared by the covalent attachment of 2,2′,4,4′,4″-pentamethoxy triphenyl methanol to a mesoporous material which further is included in a nitro resin to obtain a colourless composite. The response of this composite to diverse acid solutions was tested showing the appearance of an intense purple colour (with a colour difference higher than 160) that can be monitored by the naked eye or could be easily digitised to feed an instrumental sensor. Reversibility and resistance to washing cycles were studied with positive results. Finally, the response of the sensing composite to acid vapours was assayed, observing a colour change similar to that found in solution.

## 1. Introduction

Acids are among the chemical commodities most widely produced. They are used in the manufacture of various substances, such as solvents, chemicals, foods, fertilizers, medicines, and plastics. In fact, sulphuric acid is used in almost all industries and it is the most commonly used chemical in the world. A nation’s sulphuric acid production has been a reasonably good indicator of its industrial strength and price variations have a direct influence in the price of metals, fertilizers, grains, and other commodities [1].

Together with sulphuric acid, the most relevant acids in terms of production level and versatility are hydrochloric acid (used to clean metals, manufacturing food and organic compounds, regulate the pH level, and regenerate ion exchangers) and nitric acid (used in the manufacture of ammonium nitrate fertilizer, dyes, paints, drugs, and explosives). Other relevant acids produced in a lesser extent, but of high industrial relevance, are phosphoric, citric, acetic, formic, or oxalic acids. Furthermore, acids are used not only by industries, but also by consumers in applications such as cleaning, lime scale removal, or adjusting the pH in swimming pools.

Nevertheless, the great usefulness of strong acids has the drawback of its dangerous nature. They are corrosive substances that can cause severe chemical burns in contact with the skin or the eyes and, because they have a high vapour pressure (in particular HCl) or are able to form mists, they can be inhaled with severe lung injury. Concentrated acids can dissolve tissues easily and, therefore, cause severe damage. The corneas of the eyes are especially sensitive to hydrochloric acid and exposure to its vapours may cause severe irritation and partial or total visual impairment or blindness. Thus, it is essential that, when handling strong acids, the highest precautions are taken, using personal protection systems, such as gloves or lab coats. According to the International Labour Organization [2], 6300 people die as a consequence of work-related accidents or diseases every day. Although chemical burns are only a smaller part of such labour incidents, it must be taken into account that the accidents suffered by consumers (i.e., using cleaners) must be added to them. Despite workers, consumers lack of the appropriate education and tend to handle acids without any personal protection equipment.

Chemical burns vary depending of the country but it can comprise more than a 16% of all admission cases due to burns [3]. However, such data is based on severe cases with admission to hospitals and do not comprise minor chemical burns. According to Heinrich’s rule, for every one major injury in the workplace, 29 are minor injuries and 300 cause no injuries [4]. In China, acids are responsible for a 60–70% of the burn injures; sulphuric, hydrofluoric, hydrochloric and nitric acids being the most representative agents. Occupational settings are reported to comprise up to 67.8% of all chemical burn admissions. Although in most of the cases the exposition to acids did not cause mortality (0.6%), the average stay in the hospital was ca. 17.0 days (range 1 to 133 days), with associated economic and social costs [5,6].

The high relevance of chemical burns has focused the attention of regulators toward the development of guides and protocols to protect acid handlers, mostly in the workplace. The main strategies are based not only in the infrastructure, but also in the personal protective equipment that should be used. This includes gloves, aprons, protective shoe coverings, respirators for emergency or short-term use where high concentrations of corrosives are present in the air, face protection against splashes, etc. However, personal protective equipment is based only in the barrier properties of some materials, but lack of any smart functionalization that would allow further advice to the user about the degree and extension of an eventual exposition event.

Going beyond of the traditional esthetical and protective functions of the clothes, in recent years a new trend towards including additional functionalities in clothes has emerged and a wide set of intelligent textile materials able to interact with the human body or the environment, sense changes, and modify their behaviour has been designed. Their potential and versatility offers several applications in healthcare, military, or sport fields [7]. In general, such i-textiles are based on electronic systems constructed by the inclusion of flexible, low-power consumption systems integrated in the fibers and the clothes [8]. However, their high potential has the drawbacks of a relatively high cost and a certain complexity that reduces their practical use, in particular at home or in developing countries. In contrast, the use of optical systems has been less studied, mostly to monitor pH changes, yet not for strong acids and not focused on personal protection equipment [9,10,11,12].

Colour changes offered by chromogenic chemosensors are an interesting alternative. Naked eye response can be easily understood by end users and avoids the use of any instrument, software, or battery [13,14,15,16,17]. Furthermore, most of the indicators have a relatively low cost, opening the door to their incorporation into commodity products, such as lab coats, gloves, or other protection equipment without a relevant impact in price. Visual imaging technologies are among the most advanced and inexpensive and colour change measurement can also be instrumentalised using cameras and appropriate software.

Among the techniques to incorporate indicators into matrices, the use of mesoporous silica materials offers an interesting option. This kind of vehicle offers high surface area, increases the dye stability, reduces the lixiviation, and can modulate the dye-indicator response. Among mesoporous silica materials, UVM-7 shows particles in the micrometric range, which is an ideal size for its entrapment in a resin. There are basically two approaches for the incorporation of molecular probes into siliceous solids; surface adsorption or covalent anchoring [18,19,20]. The latter approach, although involving the synthesis of a covalently-attachable derivative of the probe, increases the stability of the dye, reducing the risk of lixiviation.

Based on our previous experience in the design of chemical-sensing ensembles based on organically-modified ordered mesoporous silica materials [21,22,23], we report herein the preparation and characterization of a pH-sensitive intelligent textile comprising an organic resin and a silica mesoporous material containing a covalently-attached pH chromogenic indicator.

## 2. Materials and Methods

### 2.1. Reagents

Anhydrous toluene, iodopropyl trimethoxysilane, acetonitrile, 2,2′,4,4′,4″-pentamethoxy triphenyl methanol (CAS number 1755-51-7), potassium carbonate, dichloromethane, ethanol, distilled water were acquired from Sigma-Aldrich Quimica, Madrid, Spain and used without supplementary purification. Calcined UVM-7 was prepared according to known procedures [24].

### 2.2. Synthesis

#### 2.2.1. Synthesis of the Dye Containing Mesoporous Material (UVM7-Dye)

**Scheme 1:**
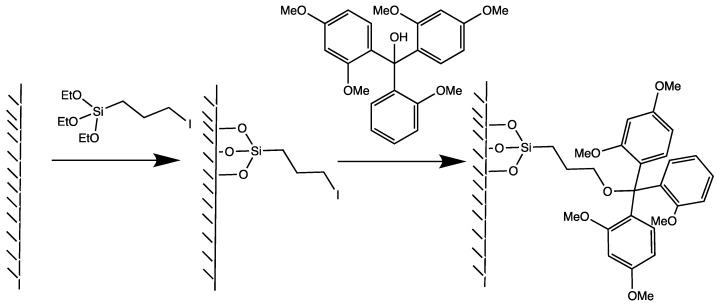
Scheme of the synthetic procedure for the preparation of UVM7-dye.

A scheme of the synthetic procedure can be found in Scheme 1. In order to prepare the mesoporous material containing a covalently-attached dye (UVM7-dye), the calcined UVM-7 was first functionalized with an alkyl iodide derivative (UVM7-I). In a typical synthesis, calcined UVM-7 (1 g) was added to a round-bottomed flask connected to a Dean-Stark trap apparatus in an inert atmosphere containing anhydrous toluene (100 mL). The resulting suspension was refluxed (150 °C), and 20 mL were collected in the trap in order to eliminate the adsorbed water by azeotropic distillation. Excess iodopropyltrimethoxysilane (1 mL, 5 mmol) was then added to the suspension and stirring was maintained for 24 h. Finally, the solid UVM7-I was filtered off, washed with 300 mL of toluene, and dried at 70 °C for 12 h.

The dye was attached to the nanomaterial through the following procedure: to 20 mL of a suspension of 1 g of UVM7-I material in acetonitrile, 2,2′,4,4′,4″-pentamethoxy triphenyl methanol (0.41 g, 1 mmol) and potassium carbonate (0.14 g, 1 mmol) were added. The reaction mixture was heated at 100 °C for 24 h under an argon atmosphere. The solid was filtered off, washed repeatedly with water, ethanol, and dichloromethane, and extracted with a Soxhlet with ethanol overnight. The solid was then dried at 70 °C for 12 h obtaining UVM7-dye.

#### 2.2.2. Incorporation of the Dye to the Textile (UVM7-Cotton and Dye-Cotton)

For the preparation of the textile containing the pigment, 10 mg of UVM7-dye were mixed with a nitrocellulose polymer and spread on a white cotton textile. The resulting material (UVM7-cotton) was dried for 24 h at room temperature. For the sake of comparison, a textile also containing only resin and the dye (in absence of the UVM-7 material) was synthesized according to the same procedure (dye-cotton).

### 2.3. Methods and Characterization

#### 2.3.1. Characterization of UVM-7 Based Materials

Thermogravimetric analyses (TGA), X-ray diffraction, and SEM techniques were employed to characterize the synthesized materials. TGA were carried out on a TGA/SDTA 851e Mettler Toledo balance, using and oxidant atmosphere (air, 80 mL·min^−1^) with a heating program consisting of a ramp of 10 °C·min^−1^ from 393 to 1073 K. X-ray measurements were performed on a Seifert 3000TT diffractometer using CuKα radiation. Scanning electron microscopy images were obtained with a Jeol JSM6300 microscope operated at 30 kV.

#### 2.3.2. Test of the UVM7-Cotton Resistance to Washing

UVM7-cotton was washed up to three times in order to test the resistance to lixiviation and verify if the textile could be compatible with conventional washing methods. In each washing cycle the textile was stirred inside water containing a commercial surfactant-based cleaning product purchased from a local retailer, followed by filtration and drying.

#### 2.3.3. Studies of Response to Acids in Solution

For the study of colour changes induced by acids in solution, HCl solutions with diverse pH in the 0 to 7 pH range were prepared (pH 0, 0.5, 1, 1.5, 2, 2.5, 3, 3.5, 4, 5, 6, 7, in the case of UVM7-dye solutions at pH 0.2, 0.4, 0.6, 0.8, 1.2, 1.4, 1.6, 1.8, 2.2, 2.4, 2.6, 2.8 were used instead of pH 0.5, 1.5 and 2.5). In a typical assay, 1.5 mL of such solutions were put into contact with 10 mg of the material (dye or UVM7-dye) or a piece of the textile (UVM7-cotton or dye-cotton). For reversibility studies, 10 mg of UVM7-dye were introduced in a solution at pH 7. The resulting suspension was moved to pH 1 with HCl observing the colour change. After, the pH was returned to pH 7 with NaOH and the colour changes were measured. A similar procedure was followed with UVM-7-cotton. The assays were performed at room temperature since it is expected that the exposure to acids suffered under ambient conditions.

#### 2.3.4. Studies of UVM-7-Cotton Response to Vapours of HCl

In order to study the response of the pigment to the presence of acid vapours, dry samples of UVM7-cotton were exposed to HCl vapours by placing them on top of a bottle for a few seconds. The colour was measured before and after such exposition. In this case, the reversibility was studied by keeping the purple textile in contact with air or under vacuum for 24 h.

### 2.4. Colour Measurement

The response of the diverse materials to acids was followed through colour changes. For this purpose, the samples were photographed with a Nikon D3000 camera, always under the same illumination and camera settings, and the RGB coordinates extracted by triplicate with the software Photoshop 10.0. Colour differences (d) were calculated with Equation (1) using the data measured at a pH in which the indicator offered no response as reference.

(1) $$d\;=\;\sqrt{{\left(\Delta R\right)}^{2}+{\left(\Delta G\right)}^{2}+{\left(\Delta B\right)}^{2}}$$

Apart of calculating the colour change, the parameter “remaining colour” (Equation (2)) was used in the washing studies. We can define the remaining colour at a certain pH as the percentage of colour change maintained after washing in comparison with no washed sample (Equation (2)):

(2) $$\mathrm{remaining}\;\mathrm{color}\;\left(\%\right)\;=\;\frac{d_{\mathrm{pH}}\mathrm{washed}}{d_{\mathrm{pH}}\mathrm{non}-\mathrm{washed}}\;\times \;100$$

In the reversibility tests, the percentage of recovery was calculated through Equation (3) (for solution) or Equation (4) (for vapours). d_pH7-pH1_ is the colour difference before and after the neutralization of the solution (pH 1 to 7), d_pH1-i_ is the colour difference before and after the acidification (pH 7 to 1), is d_t-HCl_ the colour difference between the colour measured at a certain time and the colour after the exposure to HCl, and d_HCl-0_ is the colour difference before and after the exposure to HCl vapours. Since the colour changes in the numerator and the denominator have opposite values, the absolute value of the differences is used.

(3) $$\%\;\mathrm{rec}=\;(\frac{\left|d_{\mathrm{pH}7\mathrm{ecur}}\right|}{\left|d_{\mathrm{pH}1\mathrm{ec}}\right|})\times 100$$

(4) $$\%\;\mathrm{rec}=\;(\frac{\left|d_{t-\mathrm{HCl}}\right|}{\left|d_{\mathrm{HCl}-0}\right|})\times 100$$

### 2.5. Statistical Analysis

The colour differences of the washing test were analysed by a *t* test using the IBM SPSS statistics version 24 (IBM, New York, NY, USA).

## 3. Results and Discussion

### 3.1. Sensing Materials Preparation and Characterization

2,2′,4,4′,4″-pentamethoxy triphenyl methanol, also known as pentamethoxy red, was selected as the indicator due to its chromogenic properties in response to pH. This dye is particularly suitable for sensing strong acids because it is colourless at neutral pH, but turns to an intense reddish violet colour in the presence of strong acids (pKa = 1.86) [25]. Furthermore, this indicator can be chemically attached to a siliceous matrix reducing the risk of lixiviation and ensuring that the sensing response will remain even after washing.

The indicator was fixed on a UVM-7 mesoporous material using an alkyl iodine intermediate (UVM7-I) that binds the dye through a nucleophilic substitution of the iodine by the hydroxy group of the dye resulting in the UVM7-dye sensing material (see Scheme 1). TGA studies indicated that UVM7-I contains 0.71 mmol of iodide per gram of SiO_2_, which is an amount comparable to other similar functionalized materials [23]. TGA of UVM7-dye revealed that the solid contained 0.14 mmol dye·g^−1^ SiO_2_ corresponding to a 19.5% of incorporation of the dye to the alkyl groups in the surface of the material.

**Figure 1:**
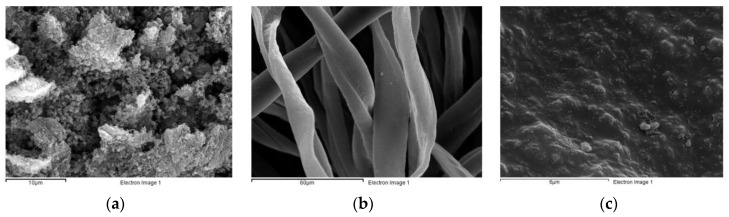
Representative SEM images of (**a**) UVM7-dye; (**b**) textile prior to modification; and (**c**) UVM7-cotton.

The XRD patterns of UVM7-I and UVM7-dye show the expected peak at ca. 2–2.5° 2θ indexed as the (100) Bragg reflection characteristic of the hexagonal ordered array of the UVM-7 material. The existence of the d_100_ peak in the XRD patterns indicated that the hexagonal ordered array was not modified during the functionalization process. SEM studies of UVM7-dye show the presence of globular microparticles formed by agglomeration of mesoporous nanoparticles. Additionally, the textile before and after the incorporation of the UVM7-dye was analysed by SEM (Figure 1b,c). Before the addition of the resin the fibres can be clearly distinguished by SEM (Figure 1b). After the addition of the sensing material the photograph shows that there is a good integration of the fibber with the resin/UVM-7 composite and that the mesoporous UVM7-dye particles are fully immersed inside the resin. Furthermore, UVM7-cotton maintains its flexibility and looks similar to the original textile after covering.

As expected, in agreement with the colour of pentamethoxy red at neutral pH, UVM7-dye and UVM7-cotton showed a colourless/pale yellow appearance after the synthesis. However, dye-cotton (prepared in the absence of the mesoporous material) exhibited the intense purple colour characteristic of the acid form of the indicator. This behaviour can be assigned to the interaction of the resin with the dye favouring the formation of a sp^2^ carbon between the three phenyl rings that extends the conjugation. This conduct indicates that the mesoporous support is a key element to maintain the functionality after the incorporation to the textile, protecting the dye from the resin. Keeping in mind the objective of developing a sensor for strong acids, we can conclude that dye-cotton was not a suitable material and was not tested further.

### 3.2. Response to Acid Aqueous Solutions

The response to acids in solution was tested with UVM7-dye (the dye attached to the UVM7 material) and UVM7-cotton (the textile covered with the resin containing UVM7-dye), in all the cases colour changes were fast, as expected in pH-induced reactions. For the sake of comparison, the pH response of pentamethoxy red in solution (PMR) was also tested. For these studies, HCl solutions with pHs in the range from 0 to 7 were prepared and pH 7 (pH with no dye response or colour formation) was used as reference for the calculation of colour differences.

**Figure 2:**
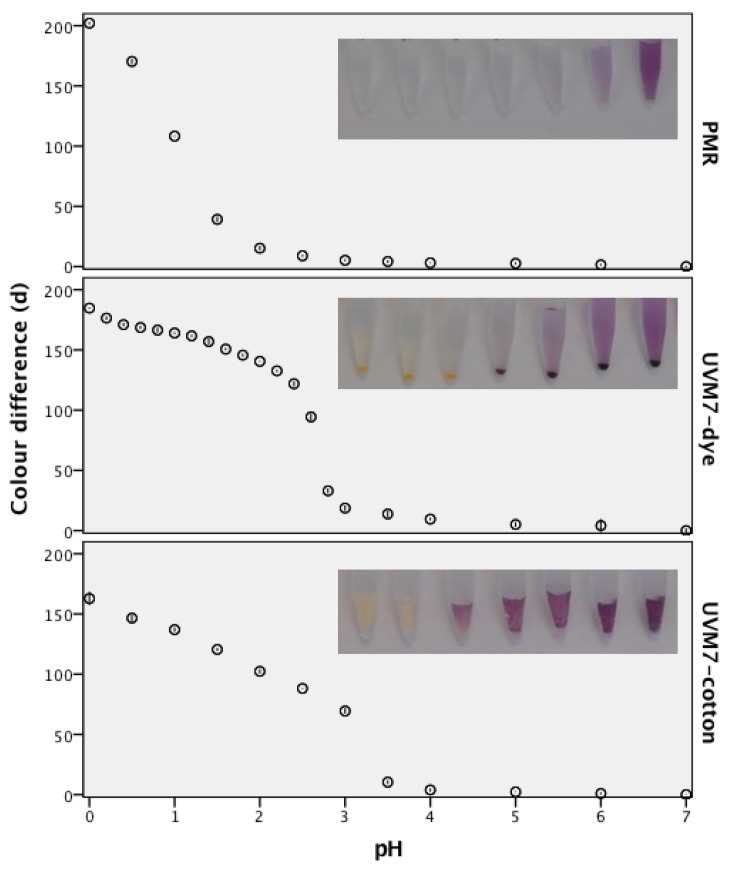
Colour differences and error bars at diverse pH for PMR, UVM7-dye, and UVM7-cotton. Inset: naked eye colour change, from left to right, at pH 4, 3.5, 3, 2.5, 2, 1.5, and 1.

As shown in the inset of Figure 2, UVM7-cotton maintains the functionality of pentamethoxy red behaving as a naked eye indicator, colourless at neutral pH, but purple in a strong acid medium, in particular at pH below 3. Colour changes at diverse pH were measured and colour differences (d, Equation (1)) reveal a variation of almost 200 units in the case of PMR at pH 0. The d value is slightly lower for UVM7-dye (d = 185) and UVM7-cotton (d = 163), suggesting that, as the support increases its complexity, the maximum colour difference decreases. However, the colour change remains intense enough to be used as a sensor and confirms that the sensing material could be coupled to an optical system and be used not only for individual protection, but also as an electronic sensor able to monitor pH, crate alerts, and notify on-line risk events.

The encapsulation affects not only the intensity at pH 0, but also the turning range of the pH (Figure 2). In the case of PMR, the free indicator in solution starts to develop colour at a highly acidic pH (<2) as expected from the pKa of the dye in the water. However, for UVM7-dye and UVM7-cotton, the encapsulation moves the turning point towards a less acidic pH and expands the turning range almost two units of pH favouring the signalling also in presence of relatively weaker acids. In order to explain such behaviour, we must consider two effects that can shift the pKa of the indicator. First, the dye attached to the UVM7 presents structural differences on comparison with PMR (in PMR is a -OH group and in UVM7-dye is an -OR group), suggesting that the formation of an alcohol moiety as a leaving group could drive the colour change to less acidic pHs. Second, mesoporous-based supramolecular systems lead to unique properties which are not simply an extrapolation of the behaviour in solution, but enhance the recognition features as a consequence of entropic factors associated with the restriction of movement, the proximity of molecular entities on the surface, and the difference of the chemical environment inside the pores in comparison with the solution [26]. We have observed previously that the inclusion of the recognition system inside the pores can be essential or increase the sensitivity of the sensors towards diverse ion orders or magnitude [23,27]. Moreover, the range is broader for UVM7-cotton (up to pH 3.5) than for UVM7-dye (up to pH 3) indicating that the resin increases a slight shift induced by the attachment to the -OR group and the porous system itself.

Figure 2 also illustrates that the support influences not only the pKa, but also induces a buffering effect, manifested in the fact that after a fast colour change, further variations as a function of pH are more progressive, i.e., for UVM7-cotton from pH 3.5 to pH 3, a colour variation (d) of 59 units is observed, but from pH 3 to 2.5 a change of only 15 units is found. In the case of UVM7-dye from pH 3 to 2.4 ∆d is equal to 103 points, but in more acidic media values of ∆d around 15 for each 0.5 point change of pH are observed.

Regarding the lixiviation of the dye, as can be seen in the inset of the Figure 2, the solution remains colourless, indicating that under the experimental conditions no lixiviation was observed. On the contrary, for UVM7-dye, a slight purple colour was observed in the solution despite being covalently attached. This indicates that, if the material is not encapsulated in the resin, upon exposition to acid the formation of the central sp^2^ carbon is able to release the dye. Although most of the dye remains in the material, a part of the dye is released to the solution. The presence of the resin reinforces the encapsulation, yet maintaining the colour change and reinforcing the stability of the dye.

### 3.3. Washing Tests

Bearing in mind the possible lixiviation of the dye, and the fact that our application is focused on the development of a smart textile able to be part of clothing or routinely exposed to water, UVM7-dye and and UVM7-cotton were washed up to three times in the presence of surfactants, and the response to solutions of pH 0, 1, 2, and 4 measured. At pH 4, independently of the sample being washed or not, no colour was developed in any sample; therefore, no remaining colour (Equation (2)) could be calculated. At the rest of pH values, as can be seen in Table 1, UVM7-dye intensity offered only minor variations that are not statistically significant for the first wash at the three pH values, or for strong acids (pH = 0), after three washing cycles. However, a significant difference (*p* < 0.05) is observed for three washes under mild acid conditions (pH 1 and 2). In any case, intensities over 98% of the original ones, even after three washing cycles, are maintained at any tested pH. This low value of lixiviation contrasts with the observation that, for UVM7-dye, part of the indicator can move to the solution in strongly-acidic media. Such behaviour reinforces the idea that the lixiviation could be induced in the acidic coloured form, but the neutral colourless form of the dye remains attached to the support and is not released during the washing cycles.

When the same experiment was performed on UVM7-cotton, a similar tendency could be observed, with a loss of colour change intensity that increases with the number of washes, and the decline of the colour intensity developed in the presence of the acid. However, the values of colour retained are in all the cases lower, in particular at pH 1 and 2 and a significant difference can be observed even after one wash at pH 2. Since we had observed that UVM7-cotton did not offer visual lixiviation of the material and we can expect a lixiviation lower than for UVM7-dye, we can suggest that the intensity of the colour change can be due to a modification of the textile properties during the washing processes. Such changes would make difficult the access of the acid to the sensing material or the accumulation of any washing substance. This effect would increase as the number of washing cycles grows and be more relevant when the concentration of acid in the solution was lower. In any case, the total retention of the colour intensity for UVM7-cotton at pH 0 was over 97%, even after three washes and it is statistically equal to the non-washed sample.

### 3.4. Reversibility Tests in Solution

**Figure 3:**
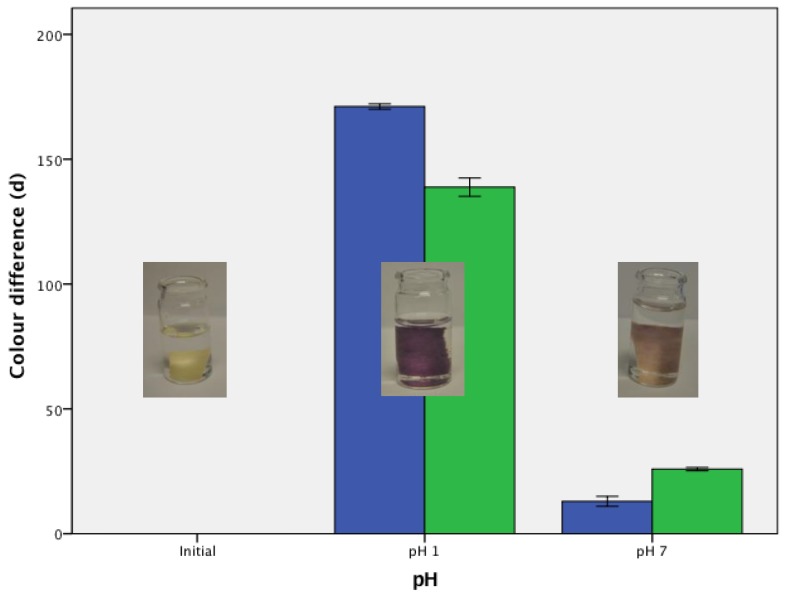
Colour differences observed during the reversibility studies in solution for UVM7-dye (**blue**) and UVM7-cotton (**green**). Inset: photographs of UVM7-cotton at the diverse pH.

In order to test the reversibility, solutions containing the sensing materials UVM7-dye and UVM7-cotton were acidified at pH 1 and later basified to pH 7. As noted above, upon acidification both materials showed an intense purple colour, but when the stimulus disappears and the pH increases progressively, the colour decreases (see Figure 3). Again, values of the colour difference around 150 are observed for both systems at acidic pH, and the colour change induced in UVM7-cotton is slightly lower than that found for the UVM7-dye in the absence of the textile.

When the pH returns to neutrality, UVM7-dye becomes colourless to the naked eye, and a recovery close to the 93% can be calculated form colour differences (Equation (3)). However, in the case of the material UVM7-cotton, although a significant reduction in the purple colour can be clearly seen, a certain colour is maintained to the naked eye (colour recovery reaches 81.4%). In the study on the response of the materials at diverse pH (Section 3.2) we observed that the colour change in UVM7-cotton was more progressive than for UVM7-dye or PMR in solution. Nevertheless, both materials offer a high degree of reversibility after acidification, opening the possibility to develop materials that can be reused after a risk event.

### 3.5. Response to Acid Vapours

The good results obtained in solution encouraged us to explore the possibility of developing textiles that could respond to acids in the gas phase, as well. Only the textile UVM7-cotton was tested because the objective was to develop a textile and the response of UVM7-dye was found to be similar to UVM7-cotton in solution. Additionally, in this case, sensing textiles with diverse washings were tested.

**Figure 4:**
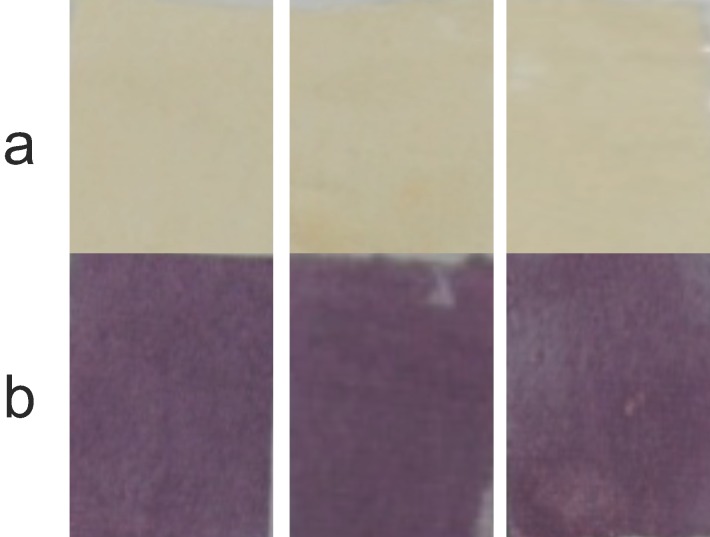
Appearance of the UVM7-cotton sample previously (**a**) and after (**b**) the exposition to HCl vapours. From left to right: zero, one, and three washing cycles.

As expected, when a dry sample of material was exposed to HCl vapours the material suffers a fast (immediate) and intense colour change from colourless to intense purple (Figure 4). Both a fast response and an intense colour change, are essential to alert acid handlers and impulse them to act as fast as possible in the case of exposition to vapours or mists of strong acids. Moreover, materials previously exposed to one or three washing cycles were found to give the same change. Measured colour differences for non-washed and UVM7-cotton samples washed one and three times display values of 166, 165, and 159, respectively, slightly higher than those observed in solution for pH 0, suggesting that vapours are able to induce a full colour change and that the sensing material is resistant to washing.

The reversibility of the interaction was studied in this case by maintaining the sensing material in contact with air, but only a minor recovery of 2% is observed after 60 min of exposition to the acid (1.35, 1.98, and 1.99 for zero, one, and three washes), which grows to 4% 23 h later (3.78, 4.12, and 4.13 for zero, one, and three washes) (Equation (4)). These low values suggest that the process is not reversible, in this case, and even maintaining the textile in contact with fresh air, the acid remains trapped in the matrix. In an attempt to force the removal of the acid, the samples were treated to vacuum, but although some additional recovery is observed (around 5% at 24 h) the results indicate that the colour change induced by the acid in the gaseous form does not reverse spontaneously and a neutralization of the solution would be necessary.

## 5. Conclusions

A pH indicator covalently attached to a silica mesoporous material has been incorporated into a textile. The resulting material is colourless in neutral media but shows an intense purple colour in the presence of strong acids, both in solution or in contact with acid vapours. The response of the material is still observed upon the application of diverse washing cycles. Furthermore, a good reversibility is observed in solution, but not in the gas phase. These properties promotes UVM7-cotton as a suitable material to be incorporated into personal protection equipment to increase safety and reduce the incidence of chemical burns caused by acids.

## Figures and Tables

**Table 1:** The remaining intensity for UVM7-dye and UVM7-cotton at diverse pH values after one or three washes, considering that the response of the non-washed material at a certain pH has a value of 100.

		% Remaining Intensity ^a^	
Material	pH	One Wash	Three Washes
UVM7-dye	0	99.75	99.04
	1	99.74	98.81 ^b^
	2	99.56	98.09 ^b^
UVM7-cotton	0	98.64	97.16
	1	98.03	92.44 ^b^
	2	96.32 ^b^	92.27 ^b^

^a^ Calculated according Equation (2). ^b^ Significantly different in comparison with the non-washed sample (*p* < 0.05).
